# The impact of the COVID-19 pandemic on mortality in Sweden—Did it differ across socioeconomic groups?

**DOI:** 10.1007/s10654-023-01068-3

**Published:** 2024-01-05

**Authors:** Thor Norström, Mats Ramstedt

**Affiliations:** 1 Swedish Institute for Social Research (SOFI), Stockholm University, 106 91 Stockholm, Sweden; 2 Department of Clinical Neuroscience, Karolinska Institutet, 171 77 Stockholm, Sweden

**Keywords:** COVID-19, Mortality, Suicide, Alcohol, Time-series

## Abstract

The characterization of the socioeconomic profile of COVID-19 mortality is limited. Likewise, the mapping of potential indirect adverse outcomes of the pandemic, such as suicide and alcohol abuse, along socioeconomic lines is still meagre. The main aim of this paper is to (i) depict SES-differences in COVID-19 mortality, and (ii) to assess the impact of the COVID-19 pandemic on suicide and alcohol mortality across socioeconomic groups. We used Swedish monthly data spanning the period January 2016–December 2021. We chose education as indicator of socioeconomic status (SES). The following causes of deaths were included in the analysis: COVID-19, all-cause mortality excluding COVID-19, suicide and a composite index of alcohol-specific deaths. SARIMA-modelling was used to assess the impact of the pandemic on suicide and alcohol-specific mortality. Two alternative measures of the pandemic were used: (1) a dummy that was coded 1 during the pandemic (March 2020 and onwards), and 0 otherwise, and (2) the Oxford COVID-19 Government Response Tracker’s Stringency Index. There was a marked SES-gradient in COVID-19 mortality in the working-age population (25–64) which was larger than for other causes of death. A SES-gradient was also found in the old-age population, but this gradient did not differ from the gradient for other causes of death. The outcome from the SARIMA time-series analyses suggested that the pandemic did not have any impact on suicide or alcohol-specific mortality in any of the educational and gender groups.

## Introduction

Socioeconomic disparities in mortality risk have been documented across a broad spectrum of causes of death. However, the characterization of the socioeconomic profile of COVID-19 mortality remains limited, with only fragmentary evidence available. Likewise, the mapping of potential indirect adverse outcomes of the pandemic, such as suicide and alcohol abuse, along socioeconomic lines is still meagre. In this paper, we use Swedish population-level data spanning from January 2016 to December 2021 to shed light on these critical issues.

The paper is organized as follows: after a brief presentation of the public health response to the pandemic in Sweden, we review the literature in the field. Next, data and methods are described. After a presentation of the findings, the paper ends with a discussion.

### The Swedish public health response to the COVID-19 pandemic

In response to the rapid increase of cases and deaths due to the coronavirus disease 2019 (COVID-19), governments and public health authorities globally launched various interventions aiming to minimize physical contacts and social interaction. In an international perspective, the Swedish strategy to handle the COVID-19 pandemic was fairly lenient, using voluntary and stepwise measures rather than lockdowns of society [1, 2]. The responsibility for preventing the spread of the virus was thus largely delegated to citizens and businesses [3]. The recommendations to control and minimize the contagion were issued on a regular basis by the Public Health Agency who had authority regarding recommendations and restrictions during the pandemic [4]. The first intervention took place on March 12, 2020 with gatherings of more than 500 people being banned; on March 29, this limit was reduced to 50 people [5]. The Public Health Agency also issued several recommendations on social distancing, personal hygiene, avoidance of non-essential travel, working from home if possible, and people over the age of 70 were asked to minimize physical contact [4, 6]. Other measures taken during the first part of 2020 were that universities and upper secondary schools were requested to teach from distance (March 17), and a ban on visits to elderly care homes was issued on April 1 [5]. A loosening of the restrictions was carried out in the beginning of June 2020 when the contagion had slowed down, and asymptomatic people were allowed to travel within Sweden [5]. All citizens were still urged to continue to take great personal responsibility and follow recommendations of physical distance. During the autumn of 2020, people were still advised to work remotely if possible. On December 14, stricter national regulations and general guidelines on individual responsibility to prevent the spread of COVID-19 were introduced, and people were encouraged to limit their social contacts during the major holidays [5]. In the early 2021, the number of COVID-19 cases increased again, which had partly to do with the new virus variants [7]. This spurred additional measures to slow down the spread of the virus, for instance by further limiting restaurants opening hours. Higher vaccination rates and a lower spread of the virus were followed by some liberalizations in the following summer, e.g. extended opening hours for restaurants and less restrictions on social gatherings (July 1) [7]. After some temporary restrictions in the fall of 2021 due to a rapid spread of infection, practically all COVID-19 restrictions in Sweden were lifted on February 9, 2022 [7].

Compared with its Nordic neighbors, Sweden had a higher incidence of confirmed cases of COVID-19, and the number of deaths was also significantly higher in Sweden [1]. The prevalence of COVID-19 cases and deaths was especially high among older and poorer residents. In addition to these direct effects of the pandemic, the restrictions gave rise to increasing unemployment, particularly in certain sectors of the economy (transport, hospitality industry) dominated by lower paying jobs [8].

### SES-gradient in COVID-19 mortality

The presence of a SES-gradient in most forms of mortality is well-established [9, 10], and there are reasons to expect a corresponding pattern also with respect to deaths from COVID-19. For instance, people were encouraged to work from home in order to reduce the risk of infection. However, it is obvious that not everyone had the privilege of this option, especially not low-SES workers. Thus, 60.1% of executives and 59.8% of professionals (with jobs requiring postsecondary education) reported working from home at least two days per week, while the corresponding number for people with typical low-SES jobs (care professions, shop assistants) was 6.3% (average for the period May 2020–August 2021 in Sweden [11]). Further, the SES-gradient in car ownership [12] implies that low-SES people are to a higher extent referred to use public transports, which is associated with a higher risk of being infected. The limited research that exists so far supports the notion that COVID-19 mortality was elevated in lower SES-groups [13]. However, much of the evidence is indirect (e.g., higher death rates in poor areas [13]), based on data from convenience samples [14], or data covering only a limited period of the pandemic [15].

### The COVID-19 pandemic, suicide and alcohol-related harm

Although the interventions implemented by authorities to prevent the spread of COVID-19 may have mitigated the physical toll of the pandemic, the potential detrimental effects of the ensuing social isolation and loneliness were highlighted early on in the public health literature; more specifically the concerns of worsened mental health [16] and increased alcohol misuse [17] were singled out. As health shocks tend to have a stronger adverse effect in low-SES groups than in high-SES groups [18], another concern is that harm related to the pandemic would be more prevalent in low-SES groups.

Suicide can be regarded as a global indicator of population mental health, and its response to the COVID-19 pandemic is therefore of interest. The pandemic may potentially have affected a host of risk factors for suicide. As noted above, although no forms of quarantine or lock-downs were exercised in Sweden, various practices and recommendations were imposed to minimize social interaction. Thus, people were encouraged to study and work from home, and to abstain from social gatherings. It seems likely that this led to more social isolation and loneliness, which tends to increase the risk for suicide [19]. Further, the job market became more precarious, with rising levels of economic uncertainty and unemployment [20], both of which are well-established risk factors for suicide [21]. Several researchers thus expressed concerns about an elevated suicide risk as a sequel of the pandemic [22, 23]. A number of studies have tackled this matter by examining the patterns of suicide mortality during the pandemic. Across various scopes, encompassing a wide array of countries [24, 25], individual nations [26], and specific jurisdictions [27, 28], the prevailing consensus derived from these studies suggests a lack of evidence of an increase in suicide rates when contrasted with pre-pandemic trends. One notable exception from this general conclusion lies in the Japanese experience [29], where a marked surge in suicide mortality was observed amidst the pandemic.

It has been hypothesized that excessive drinking could be used as a coping strategy to curb the increase of mental distress that has been witnessed in many countries during the pandemic [30–32]. Likewise, the social isolation induced by the COVID-19 restrictions may potentially have triggered alcohol misuse and alcohol-related problems particularly in at-risk individuals [17]. Aside from one German [33], and two US studies [34, 35], all reporting a marked increase in alcohol-related mortality during the pandemic, there is a lack of investigations of the impact of the pandemic on alcohol-related harm.

None of the studies reviewed above have addressed the issue of potential variations in the pandemic's effects on mental health across different socioeconomic groups. Such an approach would address the concern that the pandemic might be especially harmful for at-risk groups. Aside from the general observation, that health shocks tend to have a stronger adverse effect in low-SES groups than in high-SES groups [18], there are more specific reasons to single out the group of low-educated as an at-risk group in this context. Research based on Swedish data [36], thus suggests the presence of a SES-gradient in heavy episodic drinking with the highest prevalence among those with the lowest (primary) educational level. It seems plausible to hypothesize an elevated risk for increased excessive consumption during the pandemic among those who had a harmful drinking pattern prior to the pandemic. Further, Swedish data indicate that the group with primary education had a 6.4 times higher unemployment rate, and an almost three-fold poverty risk compared to those with postsecondary education (data for 2020/2021 from Statistics Sweden [37]).

## Aims of the study

The overall aim of this study is to examine mortality related to the COVID-19 pandemic through the lens of a socioeconomic perspective. More specifically, we will address the following research questions:

1. How large are the SES-differences in COVID-19 mortality compared to mortality from other causes?
2. Did the COVID-19 pandemic affect the number of suicides, and, if so, did the effect vary by SES-groups?
3. Did the COVID-19 pandemic affect alcohol-related harm, indexed by mortality, and, if so, did the effect vary by SES-groups?

## Data

### Socioeconomic status

We chose education as indicator of socioeconomic status (SES). We used three educational groups: (1): primary (9 years or less); (2) secondary (upper secondary school education, 10–12 years); and (3): postsecondary (college or university education, 13+ years).

### Mortality

The analyses comprised the following causes of death:

- COVID-19
- All-cause mortality, excluding mortality from COVID-19.
- Suicide
- Alcohol-specific mortality. A composite index comprising deaths with an explicit alcohol diagnosis as underlying or contributory cause of death.

ICD-codes for the causes of death are listed in Table 1.

**Table 1:** Causes of death

Cause of death	ICD9	ICD10
*COVID-19*		U07.1–U07.2
*Alcohol-specific mortality*		
Alcohol psychosis	291	
Alcohol dependence	303	
Alcohol abuse	305.0	
Alcoholic polyneuropathy	357.5	G62.1
Alcoholic cardiomyopathy	425.5	I42.6
Alcohol gastritis	535.3	K29.2
Alcoholic liver disease	571.0–571.3	K70
Alcohol poisoning	E860, E980+980	
Alcohol-induced pseudo-Cushing's syndrome		E24.4
Mental and behavioural disorders due to use of alcohol		F10
Degeneration of nervous system due to alcohol		G31.2
Alcoholic myopathy		G72.1
Alcohol-induced chronic pancreatitis		K86.0
Maternal care for (suspected) damage to fetus from alcohol		O35.4
Toxic effect of alcohol		T51
Evidence of alcohol involvement determined by blood alcohol level		Y90
Evidence of alcohol involvement determined by level of intoxication		Y91
*Suicide*	E950–E959	X60–X84

The mortality data were obtained from the National Board of Health and Welfare (Socialstyrelsen). Information on educational level was linked (by Socialstyrelsen) from the Swedish Register of Education (Utbildningsregistret) through personal identification numbers.

**Fig. 1:**
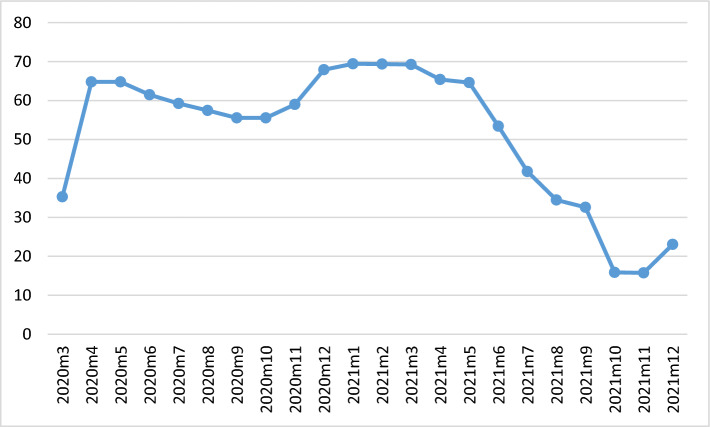
The Oxford Stringency index

We constructed age-standardized mortality rates per 100,000 (following the WHO world standard population [38] for the working-age population (25–64 years), and for the old-age population (65+ years). We chose the lower age limit (25) because the highest level of education has normally been attained at that age. All data are monthly. We used two alternative measures of the pandemic: (i) a dummy that was coded 1 during the pandemic (March 2020 and onwards), and 0 otherwise, and (ii) the Oxford COVID-19 Government Response Tracker’s Stringency Index [39], where we converted the daily observations into monthly averages (Fig. 1).

### Statistical analyses

Descriptive statistics elucidate the SES-differences in COVID-19 mortality and mortality from other causes. Excess mortality of the primary education category (indicated by RR in Table 2) was computed by taking the ratio of the mortality rate of this group to that of the postsecondary education category.

**Table 2:** Average mortality rates (number per 100,000) during the period March 2020–December 2021

Outcome	Age-group	Education	Rate	SD	RR
COVID-19	25–64	Primary	1.859	1.857	4.508
COVID-19	25–64	Secondary	0.722	0.707	1.752
COVID-19	25–64	Postsecondary	0.412	0.473	1.000
COVID-19	65+	Primary	32.610	36.272	1.657
COVID-19	65+	Secondary	25.841	29.097	1.313
COVID-19	65+	Postsecondary	19.676	22.659	1.000
Other causes	25–64	Primary	21.903	1.607	3.334
Other causes	25–64	Secondary	12.058	0.725	1.836
Other causes	25–64	Postsecondary	6.569	0.483	1.000
Other causes	65+	Primary	285.521	21.132	1.596
Other causes	65+	Secondary	233.752	17.562	1.306
Other causes	65+	Postsecondary	178.922	14.536	1.000

Rate = Number of deaths per 100,000

SD = standard deviation

RR = Rate ratio (Postsecondary education reference)

To assess whether the COVID-19 pandemic affected the number of suicides and alcohol-related mortality, we applied the technique of seasonal autoregressive integrated moving average (SARIMA) modeling [40]. Nonstationarity in the form of time trends was removed by regular or seasonal differencing. The noise (error) term, which includes explanatory variables not considered in the model, is allowed to have a temporal structure that is modeled and estimated in terms of regular and seasonal autoregressive or moving average parameters. A SARIMA model is specified as: (p, d, q) (P, D, Q, M), where the first bracket represents the model’s nonseasonal (regular) part, and the second bracket specifies the seasonal part. The order of the autoregressive parameter in the model’s nonseasonal part is indicated by *p*, whereas *d* indicates the order of regular differencing, and *q* is the order of the moving-average parameter. The symbols in the second bracket have the corresponding seasonal significance, whereas M is the number of periods per season. The noise structure was determined following established procedures [41]. Specifically, we relied on the autocorrelations and partial autocorrelations of the residuals obtained from a noise-parameter-free model to select the AR- and MA-parameters that appeared suitable. We ascertained that the residuals from the final model did not differ from white noise; this was tested using the Box-Ljung Q statistics.

The analyses of SES-differences in COVID-19 mortality and mortality from other causes were based on monthly data spanning the period March 2020–December 2021. For the analyses of the impact of the pandemic on suicide and alcohol-specific mortality we used monthly data spanning the period January 2016–December 2021. We estimated semi-log models, that is, with logged output. As noted above, the pandemic was represented as a dummy, alternatively as a continuous indicator (the Oxford COVID-19 Government Response Tracker’s Stringency Index [39]). All statistical analyses were performed with Stata V.17 (StataCorp LP, College Station, TX).

### Findings

There was a marked SES-gradient in COVID-19 mortality in the working-age population (25–64); the group with primary education had an almost fivefold risk compared to the group with postsecondary education (Table 2). The SES-gradient in mortality from other causes was less steep, with a threefold risk in the group with primary education compared to the group with highest education. The SES-gradient in COVID-19 mortality was less salient in the old-age population, and did not differ from the SES-gradient from other causes of death, with a 60% higher risk in the group with lowest compared to highest education.

**Fig. 2:**
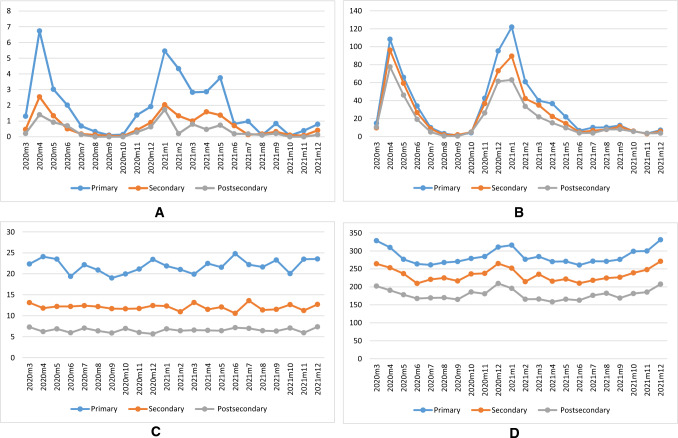
**A** COVID-19 mortality in three educational groups. Females and males 25–64. **B** COVID-19 mortality in three educational groups. Females and males 65+. **C** All-cause mortality excluding mortality from COVID-19. Females and males in three educational groups, 25–64. **D** All-cause mortality excluding mortality from COVID-19. Females and males in three educational groups 65+

The SES-gradient in COVID-19 mortality in the working-age population shows a significant monthly variation from 0.9 (August 2021) to 21(February 2021). All other months have more stable gradients between 3 and 6 (Fig. 2, Panel A). The smaller SES-differences in the elderly population range between 0.9 (October 2020) to 5.4 (August 2020) with remaining months between 1.0 and 2.6. (Fig. 2, Panel B). For comparison, trends in all-cause mortality excluding mortality from COVID-19 are displayed in Fig. 2, Panel C-D.

**Fig. 3:**
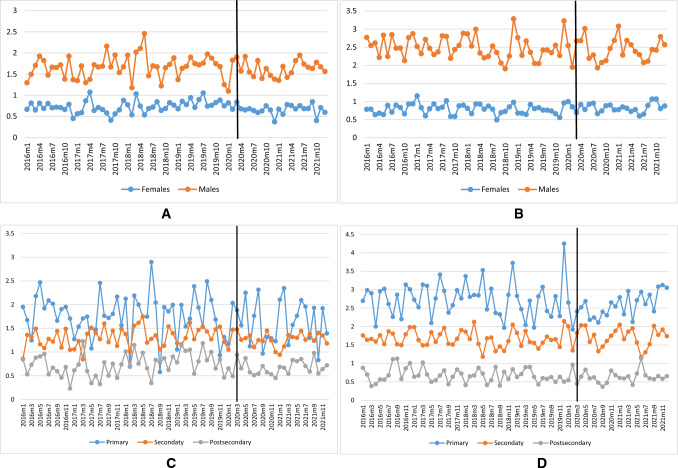
**A** Suicide rates for females (blue) and males (red) 25+. **B** Alcohol-specific mortality for females (blue) and males (red) 25+. **C** Suicide rates. Females and males in three educational groups 25+. **D** Alcohol-specific mortality. Females and males in three educational groups 25+

Trends in mortality from suicide and alcohol-specific mortality were quite stable during the study period and seem to be unaffected by the pandemic. This holds true for all educational and gender groups (Fig. 3). The graphical impression is confirmed by the outcome from the SARIMA time-series analyses which suggests that the pandemic did not have any statistically significant impact on suicide or alcohol-related mortality in any of the gender and educational groups (Table 3).

**Table 3:** Estimated effect of the COVID-19 pandemic on suicide and alcohol-specific mortality. Semi-log models. Based on SARIMA-analyses of monthly data for the period January 2016-December 2021

Outcome	Input	Gender	EST	SE	p	Q	p(Q)	Model
Suicide	Dummy	Females	− 0.031	0.268	0.907	15.215	0.230	(2,1,0)(1,0,0,12)
	Dummy	Males	0.255	0.270	0.345	9.613	0.650	(2,1,0)(1,0,0,12)
		Education						
	Dummy	Primary	0.206	0.998	0.837	12.960	0.372	(2,1,0)(1,0,0,12)
	Dummy	Secondary	0.036	0.208	0.863	15.331	0.082	(2,1,0)(1,0,0,12)
	Dummy	Postsecondary	0.411	0.635	0.517	19.871	0.070	(2,1,0)(1,0,0,12)
		Gender						
	Stringency	Females	− 0.005	0.032	0.875	15.046	0.239	(2,1,0)(1,0,0,12)
	Stringency	Males	0.018	0.030	0.559	12.883	0.378	(2,1,0)(1,0,0,12)
		Education						
	Stringency	Primary	0.040	0.058	0.497	13.861	0.310	(2,1,0)(1,0,0,12)
	Stringency	Secondary	− 0.014	0.034	0.683	8.552	0.200	(2,1,0)(1,0,0,12)
	Stringency	Postsecondary	0.056	0.062	0.366	21.256	0.068	(2,1,0)(1,0,0,12)
Alcohol-specific mortality		Gender						
	Dummy	Females	− 0.211	0.442	0.633	16.618	0.165	(2,1,0)(1,0,0,12)
	Dummy	Males	0.187	0.237	0.430	9.920	0.623	(2,1,0)(1,0,0,12)
		Education						
	Dummy	Primary	− 0.007	0.287	0.980	18.587	0.099	(2,1,0)(1,0,0,12)
	Dummy	Secondary	0.187	0.237	0.430	9.920	0.623	(2,1,0)(1,0,0,12)
	Dummy	Postsecondary	− 0.240	0.292	0.411	10.211	0.598	(2,1,0)(1,0,0,12)
		Gender						
	Stringency	Females	− 0.019	0.029	0.515	18.043	0.114	(2,1,0)(1,0,0,12)
	Stringency	Males	0.037	0.032	0.252	7.311	0.836	(2,1,0)(1,0,0,12)
		Education						
	Stringency	Primary	0.001	0.040	0.977	18.370	0.105	(2,1,0)(1,0,0,12)
	Stringency	Secondary	0.037	0.032	0.252	7.311	0.836	(2,1,0)(1,0,0,12)
	Stringency	Postsecondary	0.006	0.044	0.897	8.942	0.708	(2,1,0)(1,0,0,12)

Q = Box-Ljung test for autocorrelation

## Discussion

In this study, based on Swedish data for the period January 2016–December 2021, we found a marked SES-gradient in COVID-19 mortality in the working-age population. The risk ratio we observed (4.508) was higher than the corresponding estimate reported by Drefahl et al., 2020 (2.62) [15] pertaining to the approximate 2-month beginning of the pandemic in Sweden. This reflects the volatility of the SES-gradient which is apparent in Fig. 2. The excess risk for COVID-19 for the low-educated in the working-age group (25–64 years) was higher than the excess risk for other causes (4.508 vs. 3.334). In contrast, the SES-gradient among elderly was smaller and did not differ from the SES-gradient of other causes of death.

Contrary to concerns expressed in the early phase of the pandemic, we did not find that rates of suicide or alcohol-related mortality increased during the pandemic. This result was robust for different educational groups, as well as for men and women and independent of how the pandemic was measured. The finding that alcohol-related mortality was not affected is in line with a study of treatment seeking in southern Sweden for alcohol-related issues [42], as well as the declining trend in drinking in Sweden during the pandemic [43]. Our finding suggesting that suicide rates were not influenced by the pandemic in Sweden is consistent with the null-findings reported by most studies on this subject.

### Study limitations

The socioeconomic differences in COVID-19 were based on mortality data, and we do not know whether an analysis of COVID-19 morbidity would have led to a similar result.

Further, our focus has been on the short-term effects of the pandemic on severe forms of alcohol-related harm and mental distress. The pandemic may have had impact on less severe forms of mental distress than suicide, and on milder forms of alcohol-related harm than mortality. In addition, possible long-term effects are not captured in our study. Lastly, our findings are specific to Sweden and cannot be generalized to other countries. As noted above, the Swedish strategy to handle the COVID-19 pandemic was fairly lenient compared to most other countries, using voluntary and stepwise measures rather than a complete lockdown of society. In future research, it would therefore be of interest to compare our findings with the outcome from corresponding analyses of data for nations that implemented a more restrictive COVID-19 policy than Sweden.

## Data Availability

Request for Swedish mortality data should be approved by the Swedish Ethical Review Authority (https://etikprovningsmyndigheten.se/en/).
